# Facilitators for and barriers to nurses’ work-related health-a qualitative study

**DOI:** 10.1186/s12912-022-01003-z

**Published:** 2022-08-05

**Authors:** Dip Raj Thapa, Madhusudan Subedi, Anette Ekström-Bergström, Kristina Areskoug Josefsson, Alexandra Krettek

**Affiliations:** 1 Department of Nursing and Reproductive, Perinatal and Sexual Health, School of Health Sciences, University of Skövde, PO Box 408, 541 28 Skövde, Sweden; 2 School of Health and Welfare, Jönköping University, Box 1026, 551 11 Jönköping, Sweden; 3 School of Public Health, Patan Academy of Health Sciences, GPO Box 26500, Lalitpur, Nepal; 4 Department of Health Sciences, University West, Gustava Melins gata 2, 461 32 Trollhättan, Sweden; 5 Department of Behavioural Sciences, Faculty of Health Sciences, Oslo Metropolitan University, PO Box 4, 0130 Oslo, Norway; 6 Faculty of Health Studies, VID Specialized University, Vågsgaten 40, 4306 Sandnes, Norway; 7 Department of Public Health, School of Health Sciences, University of Skövde, PO Box 408, 541 28 Skövde, Sweden; 8 Department of Internal Medicine and Clinical Nutrition, Institute of Medicine, Sahlgrenska Academy at University of Gothenburg, Box 400, 405 30 Gothenburg, Sweden; 9 Department of Community Medicine, Faculty of Health Sciences, UiT The Arctic University of Norway, PO Box 6050, Langnes, 9037 Tromsø, Norway

**Keywords:** Health promotion, Managerial support, Job resources, Nurses, Stress, Teamwork, Work environment, Work-related health

## Abstract

**Background:**

Work-related health problems, such as work stress, fatigue, and burnout constitute a global challenge within the nursing profession. Work-related health among nurses is not yet a prioritized phenomenon in Nepal. Health-promoting approaches to maintaining and sustaining nurses’ health are therefore essential. The aim of this study was to explore and thereby gain a deeper understanding of how nurses in Nepal’s hospitals experience their everyday work, with a focus on promoting and sustaining their work-related health.

**Methods:**

A qualitative design with semi-structured individual interviews were used. Nineteen registered nurses working at hospitals in Kathmandu Valley, Nepal, were individually interviewed between October 6 and December 5, 2018. Transcribed interviews were analyzed through thematic analysis.

**Results:**

Four main themes with belonging eight subthemes were constructed from the analysis: (1) “Sense of meaningfulness and belongingness in work culture” with subthemes; “Open environment” and “Sharing attitude and cooperating for the entire team” (2) “Support and rewards from the management team” with subthemes; “Lacking managerial support” and “Fair evaluation and job promotion opportunities”(3) “Workload and protection against work-related hazards” with subthemes; “Stressful and multitasking in workload” and “Lacking equipment for own health and caring”, and (4) “Motivation through opportunities and activities” with subthemes; “Employment benefits that motivate work”, and “Activities outside of work needed to recover”. These main themes and subthemes described nurses’ facilitators for and barriers to their work environment and health.

**Conclusion:**

Our study highlighted nurses’ experiences with facilitators and barriers to their work-related health. Nurses’ work-related health was positively affected by support from colleagues, managers, and the organization. Conversely, less support from managers, lack of equipment, and unfair judgment were barriers to nurses’ work-related health. This study adds new knowledge about nurses’ work-related health from the context of Nepal. Hospital organizations and nursing managers in similar cultural and healthcare settings can apply the results of our study to develop strategies to promote and sustain nurses’ health and prevent work-related illness.

## Introduction

The nursing profession is devoted to the care of people and theworldwide promotion of health [1]. However, it is associated with several work-related stressors, such as organizational service, care-related issues, organizational roles, and colleague-related issues [2]. The key job demands of nursing staff are evident as work overload, lack of formal rewards, and work-life interference [3]. Work-related stress and burnout are prevalent and negatively affect nursing professionals’ health [4]. Sick leave due to work-related stress is highly prevalent in high-income countries, especially those in the European Union [5], and not only causes illness but also increases the economic burden on society [6]. Among nurses, work-related health challenges are increasing, even in low-income countries. For instance, moderate to high job-stress levels are prevalent among nurses in Nepal [7], India [8], and Bangladesh [9].

Aside from their professional responsibilities and challenges, nurses frequently confront care-related ethical dilemmas [10], which can cause them moral distress in complex healthcare situations [11]. Consequently, the nursing profession is especially vulnerable to work-related health challenges. This, coupled with an ongoing global shortage of nurses [12] and a growing aging population with increased care demands [13], imposesan extensive burden on nurses. These challenges are accompanied by—and likely contribute to—an increasing turnover rate among nursing professionals that is, in turn, adversely impacting the health outcomes of both staff and patients [14].

There are various professional resources that can help nurses to manage their workload and stress. For instance, a recent study showed that supervisory support, interpersonal relationships, and transformal leadership are among the key resources for employees [3]. Similarly, workplace social support and appropriate work–life balance can also enhance job performance and lead to stress reduction [15]. For nurses specifically,a meaningful work context andcomprehensible and manageable health resources can bolster job satisfaction and thereby reduce staff turnover [16].

Exploration and identification of work-related health barriers (demands) and available facilitators (resources) are necessary to promote and sustain nurses’ work-related health. This becomes more important in the context of Nepal, where work-related health has not been prioritized and there is limited research on the issue.

## Background

The World Health Organization (WHO) defines work-related stress as an imbalance between work demands, work pressure, and employees’ knowledge and ability to cope. Therefore, employees need to perceive work-related stress as manageable and remain motivated and alert to manage their stress [17]. The Ottawa Charter for Health Promotion emphasizes the importance of cultivating the capacity to cope with stressors and exercise control over and improve health [18]. Antonovsky introduced a similar concept through the theory of salutogenesis [19]. In this theory, a resource perspective is considered vital to maintaining and promoting health, as it provides solutions to perceived problems. Antonovsky emphasized that stressors are unavoidable in many ways, and that, through coping strategies, individuals can manage their stress and well-being [19].

According to such reasoning, workplace stress can be seen as positive, provided it can be managed through appropriate and sufficient resources. Indeed, job-related burnout is the result of excessive job demands due to resource deficiencies [20]. On the other hand, when job resources are both readily available and sufficient, they can increase work motivation and engagement [14]. The job demands–resource model has established the concept that employees’ job performance and health outcomes are related to bothjobs demands and job resources in the work environment. High levels of jobdemand, such as high workload and work pressure, may lead to emotional exhaustion and burnout; however, job resources protect health, stimulate personal growth, and motivate employees for better work engagement and job satisfaction [21, 22].

Research on stress and coping among nursing students in Nepal started in the 1990s by specifically measuring coping behaviors and potential solutions to stress-related problems. The research identified as stressors a lack of interpersonal relationships as well as inexperience among newly graduated nurses, which generated the perception and experience of a heavy workload and feelings of helplessness [23]. Although most nurses in Nepal’s hospitals are satisfied with their work, someperceive a lack of support in their daily work, which combined with their inexperience,negatively influences health [24]. Conversely, asupportive working environment with warm and welcoming behaviorshas a positive impact on workplace health [25]. High or moderate stress is experienced by 12% of nurses in tertiary care units in Nepal, whereas 62% of nurses working in critical care units perceive moderate to high levels of work-related stress [26]. Recently, Gurung et al. [27] found that 66% of nurses in government hospitals and 79% in private hospitals in Nepal experience moderate to severe job-related stress.

The current prevalence of work-related health problems among nurses in Nepal is mainly presented in quantitative studies and data on in-depth experiences of the overall work environment and its significance for nurses’ health in Nepal is limited. More research is therefore needed to determine the present health status of nurses and document their perceptions of and experiences with work-related health challengesin Nepal. Increased understanding of nurses’ work-related health is vital to ensure the development of effective methods for maintaining and sustaining their health.

## Methods

### Aim

The aim of this study was to explore and thereby gain a deeper understanding of how nurses in Nepal’s hospitals experience their everyday work, with a focus on promoting and sustaining their work-related health.

### Design

We employed a qualitative design with semi-structured, in-depth, open-ended interviews. The design was guided by an inductive approach to document nurses’ experiences concerning their work-related health. The open-ended dimension of the interviews gave the participants the opportunity and flexibility to describe and reflect upon their perceptions and experiences in their own way.

### Setting

We conducted our study in Lalitpur and Kathmandu, which are both metropolitan areas in Nepal’s Kathmandu Valley. They are situated in the central-east region of Nepal, which includes some of the country’s most populated cities [28].

The nursing profession includes several work categories in Nepal, where there are currently 67,075 registered nurses and 845 foreign nurses [29]. The nurses have different educational levels: Proficiency Certificate–level (PCL) nursing is a basic nursing level that involves three years of direct education after Grade 10. Nurses with a Bachelor of Nursing Science (BNS) degree receive three years of additional education after obtaining their PCL certificate in nursing. A Bachelor of Science (BSc) in Nursing involves four years of education with science as a major. This education can be undertaken only after completing Grade 12 [29]. An abundance of job opportunities and available career openings has led to increased interest in nursing education and the nursing profession in Nepal [30]. We chose Kathmandu Valley for our research because it is where most private and government teaching hospitals, and nursing homes are located, due to centralization.

### Participants

Nineteen female registered nurses were included for participation in the study. The inclusion criteria were as follows: being a registered nurse, not being on sick leave, speaking either Nepali or English, working full-time between 75 and 100% as a nurse, and having a minimum of one year of work experience.

### Data collection

#### Sampling methodology

We recruited nurses via purposive sampling with a snowball strategy. The second author established initial contact with the departmental managers of two hospitals and mediated these contacts with the first author. The department managers then contacted potential participants. Each potential participant, once recruited, was then asked to contact friends or colleagues who worked as nurses in different departments of the same hospital or at another hospital.

#### Recruitment procedures

The first author contacted the participants, who chose where and when to meet. We obtained informed consent, both verbally and in writing, before proceeding with the interviews. All participants were informed about the nature and purpose of the study as well as the interview process. Similarly, we ensured the participants’ anonymity and confidentiality. We also ensured, orally and in writing during the initial in-person meeting, that the participants were aware of their right to withdraw from the study at any time, with or without explanation. We selected five different hospitals (three public hospitals and two with mixed private and public ownership) and strategically recruited participants from different departments for the purpose of variation. Participants who verbally consented to participate in the study were contacted by phone by the first author to schedule a date, location, and time for the interview.

#### Interview procedures

The first author, who is originally from Nepal, conducted the interviews between October 6 and December 5, 2018, in the Nepali language. The interviews were performed outside the hospitals, in quiet roomsat nearby restaurants chosen by the participants. Separate locations were required to ensure that the interviews remained confidential. Theywere conducted based on an interview guide containing questions related to nurses’ work environment that focused on how their health could be promoted and sustained. We developed the interview guide based on our previous research (Ref. anonymized) and pretested it through two pilot interviews, which were subsequently evaluated. Pilot interviews were transcribed and translated directly after the interviews. Discussions regarding the interview guide, research question, and the responses (experiences) were done firstly with the first and second authors and then sent to all co-authors for feedback. We determined that the interview guide sufficiently covered the research area and was able to answer our research question. We therefore included both pilot interviews in the final analysis. The interview began with a warm-up question: “What does your daily work routine look like and can you please describe your everyday work.” This question enabled the participants to think about their everyday work tasks, and it was followed by the opening question: “Can you please describe a situation that you have experienced in your workplace that had a positive or negative impact on your health?” The participants were permitted to freely think about and reflect upon their experiences. The open questions during the interview were complemented by follow-up comments and questions, such as “Please describe your feelings about the situation in more detail” and “Please explain how you handled the situation.” The follow-up questions encouraged the participants to describe their strategies and solutions concerning existing health challenges in their workplace. The interviews lasted between 20 to 50 minutes, were audio-recorded, and were transcribed verbatim in Nepali language and translated directly into English by a professional translator. The interviews ended when the interviewing author determined that data saturation had been reached. Data saturation was reached when the interviewing author experienced that the information provided by the participants was mostly repeated from what earlier respondents had shared [31]. A discussion among co-authors about data saturation was made to confirm that the provided experiences through 19 interviews were sufficient to answer our research question. In all, the transcribed interviews resulted in 178 pages of text (A4).

### Ethical considerations

We followed the ethical guidelines as stated in the Declaration of Helsinki [32]. Ethical permission to conduct the study was obtained from the Nepal Health Research Council (Ref. 684–2018). Information about the study, including its voluntary nature, was provided to all participants. Written and verbal informed consent was obtained from each participant before the interviews began. To ensure the confidentiality of all personal data,each interview was coded with a specific number to prevent the identification of the participant. All background information connected to the interviews was saved separately and securely stored. To further safeguard the participants’ confidentiality, the results have been presented in such a way that no personally identifiable information is included. The participants were given no incentive for participation,although they were provided coffee and snacks after completing the interview.

### Data analysis

We thematically analyzed the open-ended interview questions with an inductive approach [33]. The thematic analytical process involved six steps and followed a practical guide for thematic analysis developed by Braun and Clarke [34]. The coding and creation of themes followed the latent level of analysis, while the coding and creation of subthemes followed the manifest level of analysis. In the first step, we listened to the recorded interviews, read through the transcripts, and then re-read the transcripts to ensure familiarity with the data. In the second step, we marked, sorted, and coded all relevant meaning units/text/content related to the aims of the study, with the meaning units most pertinent to addressing the aims of the study entered into an MS Excel file. We employed open coding, meaning that we could modify and develop the codes at any point during the coding process. In the third step, we identified preliminary themes by comparing different codes and marking those with similar meanings/content with similar colors. We assigned names to the preliminary themes that described the essence of the codes and the data, and we also identified overlapping themes. We created subthemes from the largest codes that we could match with the main themes.

In the fourth step, we reviewed the identified themes. In this step, we read and re-read texts, codes, subthemes, and themes and compared them with respect to our research aims. When overlapping information was identified in the themes or subthemes, we moved the codes to more appropriate themes to eliminate the overlaps. Similarly, we revised the names of the themes and subthemes until we were satisfied that they were appropriate with regard to the content of the transcribed texts. In the fifth step, we defined the final names of the main themes and associated subthemes (see Table 2). At this stage, for the purpose of addressing the research aims, we discussed whether the themes and subthemes had any overlaps and whether other themes and subthemes were interrelated. In the last step, we generated the finalized results. An example of our analytical process is presented in Table 1. To illustrate the subthemes, we relied on quotations from the interview transcripts. The first author initially created the codes, whereas the second author carried out the procedures in the second step and was responsible for finalizing and documenting the themes and texts. The other authors were responsible for reviewing the codes, themes, and content of the transcribed texts.

**Table 1:** The thematic analysis process from text to themes and sub-themes

Meaning units/texts	Code	Subtheme	Theme
When others share when they are stressed I feel like it is not only me. There are many others too in the same kind of situation. We can also identify problems if many share the same kind of problems - it is easier to find solutions	Openness and sharing between colleagues help identify problems and solutions	Open environment and sharing attitude	Sense of meaningfulness and belongingness in work culture

### Rigor

The interviewingauthor’s reflection about “positionality” has been important for the study’s trustworthiness. Now a male registered nurse and doctoral student residing in Sweden,that author was born and raised in Nepal and is therefore knowledgeable about both its language and culture. He could therefore engage the nurses to share their experiences in their own language, which ensured the study’s credibility. Due to the hierarchical organization and male-dominated nature of Nepali society, a power imbalance may have been present during data collection, as all of the participants were women. To rectify this, the interviewing author sought to foster an open climate that would encourage the participants to share their experiences. A face-to-face appointment with the participants before the interviews increased the sense of comfortable communication during the interviews. The second author is from Nepal, and additional Swedish co-authors have expertise in nursing, public health, global health connected to research in Nepal, occupational health, and qualitative methodology. Furthermore, the co-authors designed and shaped the study, and participated in the analysis process which contributed to the construction of subthemes and themes. The combined competencies of the authors ensured broader perspectives in the analytical process. This led to reflexivity about the authors’ positionality beingunderscored during data collection and analyses, which strengthened the trustworthiness of the study [35].

We used the consolidated criteria for reporting qualitative research (COREQ) [36]. Applying these criteria contributes to the validity and transferability of our results [36]. We also employed established criteria for trustworthiness, as described by Elo et al. [37], who asserted that the trustworthiness of a study is determined by the extent to which the researchers sufficiently and clearly reflect upon the research process in its entirety. Trustworthiness also entails the degree to which the quality of methods, interpretations, and presentations of the data has been ensured [38]. The analytical process from coding and establishing themes and subthemes involved discussion among all authors, thus contributing to the confirmability of the study. As the same author conducted the interviews and initiated the analysis,the dependability, and thus the trustworthiness of the research, was strengthened [39].

## Results

### Demographic information of the participants

The participantsranged in age from 24 to 53 years old (mean age of 31.8 years). Their work experience ranged from four years to 30 years (mean work experience of 10.9 years). Two of the participants had a master’s degree and 17 had a bachelor’s degree. Fourteen of the participants were married. Participants worked at different wards in the selected hospitals; surgical ward, orthopedic ward, maternity ward, endoscopy ward, pediatric ward, psychiatric ward, emergency ward, as well as labour and post-natal ward. All participants resided in the Kathmandu Valley, and they worked in different wards at the selected hospitals.

### Main findings

Four main themes were constructed from the interviews:“Sense of meaningfulness and belongingness in work culture,” “Support and rewards from the management team,” “Workload and protection against work-related hazards,“and “Motivation through opportunities and activities.” In addition, eight subthemes were constructed from the main themes. An overview of the main themes and subthemes is given in Table 2.

**Table 2:** Overview of themes and subthemes

Themes	Subthemes
**Sense of meaningfulness and belongingness in work culture**	Open environment and sharing attitude
	Cooperating for the entire team
**Support and rewards from the management team**	Lacking managerial support
	Fair evaluation and job promotion opportunities
**Workload and protection against work-related hazards**	Stressful and multitasking in workload
	Lacking equipment for own health and caring
**Motivation through opportunities and activities**	Employment benefits that motivate work
	Activities outside of work needed to recover

#### Sense of meaningfulness and belongingness in work culture

The main theme of a sense of meaningfulness and belongingness in work culture comprises two subthemes: “open environment and sharing attitude” and “cooperationacross the entire team.” These subthemes explained the nurses’ experiences of their work environment and team cooperation in their everyday work. The nurses considered their work environment as meaningful. Their colleagues influenced and motivated them to be productive and to feel satisfied and healthy at work. Additionally, they experienced harmony, belongingness, and meaningfulness in their work.

##### Open environment and sharing attitude

Open work environment and sharing attitude were considered to be facilitators for nurses’ health and work. The nurses viewed their work environment as a platform for sharing, learning, supervising, and supporting each other. Most of the nurses found their team to be supportive and encouraging of each other when needed. They also considered their work to bean open environmentin which sharing with colleagues was common. They felt theycould resolve problems, address challenges cooperatively, and help each other with both administrative and care-related responsibilities. Through such cooperation, the nurses felt cared for by each other:

*All of us share our opinions. When we communicate well, it feels good. Sometimes, information is related to the patient; sometimes it can concern our homes. Sharing can be about homely events as well as treatment progress or the patient’s background.* (Nurse 2)

An open work environment also made it easierfor the nurses to sharetheir feelings and difficult experiences,such as when something was missed or handled wrongly. The nurses could directly share their feelingsabout a situation and therebyobtain relief from the associated stress. An attitude of openness prevailed, one which permitted the nurses to freely ask about care-related issues andlearn from others to solve problems as they arose. The nurses believed that this prevalent attitude ofsharing contributed to feelings of belongingness and increased their attachment to each other.

##### Cooperating across the entire team

Cooperation in the team was viewed as an important aspect of nurses’ work environment and health. The nurses regarded teamwork among colleagues as one of the most important elements of their daily work,with sufficient levels of cooperation serving as the basis for success in their workplace. Almost all of the participants were satisfied with the cooperation they received from their colleagues. Work satisfaction was obtained through the strong bonds they shared within the team. However, some participantsfelt dominated by the doctors/supervisors in charge of their ward, with some adding that they felt insulted and intimidated by the doctors’ behavior.

*Sometimes, there are weaknesses related to hospital organization and the doctors know about that. Sometimes doctors give orders in front of visitors. They say, “sister, you should have done this, and I had told you to do it that way”. They act as if they are superior.* (Nurse 11)

It was important for the nurses to feel they had the freedom to ask about the challenges that arose in their daily work. Typically, however, only when a nurse had been working for some time could she cross boundaries, such as asking questions of colleagues at higher hierarchical levels. A sense of togetherness was also expressedthrough mutual appreciation. Being together and supported by colleagues resulted in decreasedlevels of stress. This was especially helpful when the workload was high, as the nurses could divide tasks among themselves and support each other.

*We work as a team: The ward itself is a complicated ward, so it is ok. We have started to adjust. We adjust through our cooperation. We divide work among ourselves. We prioritize,so it is easy.* (Nurse 3)

Due to strong cohesion among the nurses, theyfoundthe workplace to be more important and pleasurable than their own home. The effectiveand efficient cooperation and emotional support cultivated in the workplace increased the nurses’ sense of comfort and helped themfeel like essentialmembers of the team, in turn generating stronger emotional support among them.

*Yeah, it is due to the good friends and good support from them, that I am still working ( … ) I feel good. I feel my ward is my home.* (Nurse 8)

#### Support and rewards from the management team

The main theme ofsupport and rewards from the management teamincludes two subthemes:“lacking managerial support”and “fair evaluation and job promotion opportunities.” The main theme described nurses’ experiences of support from the management teamin their everyday work. Increased administrative responsibility and insufficient rewards were noted as factors that negatively affected the nurses’ health. Having a management team that provided timely and sufficient support as needed and which evaluated nurses’ work fairly was considered important for dealing with daily work-related stressors.

##### Lacking managerial support

Nurses’ experienced their managers’ support as an important factor in their daily work, however, they were lacking enough support and guidance from their managers to handle daily work activities. The nurses were required to take on both administrative and care-related work on their own. Nevertheless, some of their work tasks could not be done without the help of managers. Management-related responsibilities, such as ensuring the proper functioning of care-related equipment, were expected of the nurses, often on their own. During periods when workloads were heavy, such as when there was an overflow of patients, nurses were expected to work overtime without payment. Sometimes, the nurses did not ask for help because they feared that uncompleted tasks could be interpreted as incapability or incompetence.

*Even working for an extended period during the shift is normally taken negatively. The manager usually says that she could not finish her work in time,so she is staying for extra time. She could not finish her work on time. They may feel as if she is unable to work, or she could not manage well*. (Nurse 2)

Even when managers were helpful, the nurses would still experience a lack of support when they needed it. Additionally, there was a gap in the relationship between managers and nurses, likely attributable tothe much higher hierarchical level of managers compared with that of nurses. As such, nurses were expected to listen totheir managers and comply with their instructions. Most work responsibilities were carried out within thework teams. The nurses also experienced a lack of support when managing administrative responsibilities, such as shift changesand finding substitutes when theywerescheduled for leave. Such lack of managerial support resulted in feelings of frustration and increased workload. The nurses claimed that managers perceived them to be complaining when they asked for help and support, and consequently the nurses were reluctant to seek assistance from their managers.

*Whenever I need my holidays or if I need to leave, I need to ask her right? But I feel awkward to ask. Instead of giving suggestions, she keeps scolding and says I will take you to matron because I am asking for leave. Therefore, we are afraid to ask.* (Nurse 12)

##### Fair evaluation and job promotion opportunities

Being fairly evaluated and promoted for higher job positions were important for nurses’ job motivation and could affect their well-being. The prospect of receiving a job promotion was an important motivating factorfor nurses in their work,especially when it involved moving up the hierarchical ladder. Being consistently successful and diligent at work increased the chance of promotion, thereby climbing the organizational ladder, and as suchthe nurses sought to do their best to be promoted. The nurses consideredbeing judged and evaluated well and obtaining a promotionextremely valuable. Theyalso expressed how hard they worked,so that it would be clear to their manager. However, the nurses found evaluations were lackingin fairness, particularly as there were no clear criteria for judgment. Work evaluations and promotions were often decided by higher-level managers, and the nurses were not able to determine why some of their colleagues were promoted and others were not. Such ambiguity and absence of clear judgment criteria concerning work performance and promotion caused significant stress and dissatisfaction among the nurses, which impacted them negatively.

*I should have been promoted to the seventh step after seven years of being promoted. There was no complaint from the ward, and I was good in terms of doctors, and evaluations too. But still, there was a delay of about one and a half years for my promotion. When asked for a cause or reason, nobody gave me a reason. So, because of that, I got tense. I even thought of leaving the job.* (Nurse 13)

Being acknowledged, appreciated, and rewardedwas important for nurses’ motivationto do their work. However, most lacked rewards orsigns of appreciation from their managers or doctors. Since doctors were higher in rank and decided on treatments, it was also important for the nurses to be appreciated by them; however,their ideas were not taken as input and were often neglected. Some nurses equated cooperation with managers and doctors with more responsibility and increased work burden.

*Some doctors shout at junior staff and dominate them. They just complain because they are new but if they see senior staff, they speak politely. The voices change accordingly.* (Nurse 4)

#### Workload and protection against work-related hazards

The main theme ofworkload and protection against work-related hazardscomprises two subthemes: “high workloadrequiring multitasking” and “lacking equipment for own health and patient care*.*”The main theme described nurses’ experiences concerning workload and protection in their daily work. A manageable workload was important for their recoveryand the nurses found being protected from work-related hazards was indispensable for feeling safe and protected from communicablediseases.

##### High workload requiring multitasking

High workloads and multitasking were important health barriers for nurses and affected their health negatively. Most nurses found their work to be stressful, with high workload and frequent multitasking. In particular, the number of patientsper nurse was seen to be difficult to manage and unreasonable and resulted in the nurses feeling that they were overloaded with work. The number of staff remained fixed even when the number of patients increased, sometimes dramatically so. The nurses felt that cooperation and the division of work tasks among colleagues were the only effective solutions. Additionally, a high patient ratio increased the likelihood of patients receiving a lower standard of care.

*Next thing is that there is a more patient flow here. We need to receive patients beyond our capacity. We have 33 beds, but we have more patients than that. Sometimes, we put them in trollies and sometimes even on the floor ( … ) We cannot talk to and deal with all the patients.* (Nurse 17)

Multitasking meant taking care of almost all types of work responsibilities, which led to a heightened work burden. The nurses felt responsible for fulfilling patients’ basic needs, providing information, and explaining the health and care of patients to visitors, including family members. Similarly, the nurses were obliged to assume administrative responsibilities, such as moving patients to other wards and ensuring that all care-related materials were available. They emphasized how challenging it was to complete all of these tasks, sometimes simultaneously, while at the same time taking care of visitors. The nurses often had to explain the same thing several times to different visitors, which was frustrating and generated increased stress and irritation. Worse, some visitors were often difficult and combative.

*Visitors always feel that the patient is sicker than other patients. We must prioritize; but for some, they feel as if their patient is more complicated than other patients and we didn’t take care of them. I feel sad. We cannot look after all patients at the same time.* (Nurse 5)

The high workload resulted in feelings of being incapable of providing sufficient care for patients,which made the nurses sad and frustrated. They were also often exhausted fromworking long hours without sufficient time for rest and recovery. The adverse health effects of high workload included headaches, loss of concentration, irritation, sleep deprivation, inadequate recovery, gastritis, and leg pain.

Many nurses noted that the amount of stress they experienced was directly related to the types of the patient for whom they cared. For instance, caring for patients in the emergency ward, psychiatric ward, maternity ward, or pediatric ward was considered far more challenging and stressful. The nurses also remarked that some groups of patients, such as those with drug and alcohol problems, were more vulnerable and difficult, leading tomore severe care-related problems.

*… but yeah, at the time of rush we get really tired and cannot sit or rest. Sometimes, there might be drunk patients and you may need to quarrel. It is not easy to work like before. You need to be very cautious before speaking. So, there is stress about certain things. Sometimes, there are quarrelsome patients that do not follow the guard or the police. It is stressful at that time.* (Nurse 14)

##### Lacking equipment for own health and patient care

Nurses’ health was negatively affected due to the lack of equipmentneeded to protect themselves and patients in daily work. One of the most important aspects of the work environment for the nurses was feeling safe from work-related hazards. Being a nurse means dealing with different types of patients suffering from various conditions, especially those with communicable diseases. Workplace injuries, such as stabbings, were also common. The nurses lamented that they lacked the proper equipment to protect themselves. Consequently, they were afraid of contracting diseases and infections. Sometimes, equipment crucialfor protecting patients was missing, and yetthe management team was often not capable or even unwilling to provide such equipment. The nurses felt responsible for protecting their patients and themselves from all types of hazards. Some nurses stated thatthey had to bring masks and gloves from home, as they were in short supply at work or were unavailablealtogether.

*You know there are exposures, and we know there are exposures, and to protect us from the exposures, we take materials from home, such as masks, you know.* (Nurse 1)

One fear expressed bythe nurses was unknown patient health history. They often cared for patients who came from far away and did not have any healthcare documentation. As many patients lacked proper identification, the nurses were forced to trust what the patients or their families told them. Many patients could not explain that they had contracted an infectious disease,or sometimes were not even awareof having done so. Therefore, the nurses were frequently left with persistent feelings of uncertainty and fear that they could contract an infectious disease.

*We do not have time to screening patients’ documents and we do not have time to use protective clothing. There are hazards and there are risks. There is so much rush. Therefore, we are at risk of getting serological diseases like HIV.* (Nurse 15)

#### Motivation through opportunities and activities

The themeofmotivation through opportunities and activitiesinvolves two sub-themes:“employment benefits that motivate work” and “activities outside of work needed for recovery*.*”Opportunities and facilities provided by their employer, as well as activities geared toward facilitating recovery,were noted by nurses as important sources of health and well-being.

##### Employment benefits that motivate work

The nurses were motivated to work by different facilities and opportunities provided by their employer which in turn affected nurses’ health positively. Being a nurse was a source of pride for the participants. They emphasized that their primary motivation to work was caring for others and receiving benefits from work. Receiving feedback from patients, especially positive feedback, strengthened their resolve. Concerning benefits, the nurses received medical insurance and free regular medical checkups, and 90% of medical treatment costs for their family members were covered as well. In addition, the nurses felt independent because of their work and were happy that they couldshare their benefits with their families. In this respect, the nurses felt humbled and were gratefulto their employer.

*Because of the facility of medical insurance in the hospital, we have freemedical checkups and discounts for various investigations, not just individually but also for our families, including my parents, husband, and two children. I think that is a big thing in the context of Nepal. That’s because contrary in other nations where the government looks after the health of citizens, that is not the case here in Nepal.* (Nurse 13)

The nurses also expressed that the skills development training and educational training provided by their employer played an important role in safeguardingthem and providing good care to patients. The workplace was perceived and experienced as being a source of knowledge that increased nurses’ sense of security and positive feelings at work. Being a nurse means being able to care for others and also increasing health literacy for oneself. Having sufficient care skills also entails being alert to and gaining knowledge about the different health problems and diseases that nurses should be aware of. Possessing such skills strengthened them both individually and professionally.

*Yes, there will be doctor’s contact. I will also have knowledge, knowledge about many diseases. I think it is due to knowledge. When one has knowledge, he/she knows about what is happening in advance and possible symptoms which makes it easier to treat.* (Nurse 6)

##### Activities outside of work needed for recovery

The nurses adopted different strategies, including social and physical activities, to recover from work stress. They mostly relied on activities such as meditation, listening to music, visiting family and friends, and performing yoga, all of whichassisted in their recovery. Their employers offered educational workshops, which werealso appreciated by the nurses. In addition, many social activities were often arranged outside of work, in the nurses’ free time, and these were found to be just as important for recovery from work-related stress.

*Then we arrange a get-together, outing, or picnic to refresh ourselves. Recently, all of us went to the zoo, two or three days ago. If there is a continuous workload, we plan for a gathering at my own house, or we visit any place.* (Nurse 8)

Long working hourswere considered harmful and caused leg and back pain in some of the nurses. However, most considered physical activity during work to beadvantageous for their health. They had generally limited planned physical activities;therefore, walking during work was considered an important source of physical activity. Physical work was considered to be generally positive since the nurses’ minds were occupied with solving care-related problems. Physical activity thus reduced their experience of stress.

*There is physical activity at work. The mind is occupied, and so, if there is any stress factor at home, it gets diverted due to work.* (Nurse 17)

## Discussion

We explored facilitators and barriers tonurses’ work-related health through their own experiences of everyday work at hospitals in the Kathmandu Valley, Nepal. For the most part, nurses found their work to be meaningful, with good teamwork and an attitude of sharing prevalent among their colleagues. Support from the management team was valuable, although the nurses did not experience such support sufficiently and were not fairly evaluated for their work. Multitasking, increased administrative responsibilities, and a shortage of staff resulted in work overload and significant stress. Lack of equipment and materials resulted in concerns for their own health and that of their patients. Being employed as a nurse and the benefits such employment confers, such as insurance, were motivational factors for the nurses. They adopted social and physical activities as strategies to recover from their work-related stress.

The nurses in our study experienced meaningfulness and belongingness in their work culture, both of which played an important role as facilitators in their daily work and work-related health. An open environment with a widespread attitude conducive to sharing among the nurses and other colleagues strengthened and motivated them to work. Through cooperation and teamwork, the nurses were able to resolve most of their daily patient-related and administrative work tasks. In addition, they experienced a sense of togetherness, which enhanced their job motivation and satisfaction. A recent study from India yielded similar results regarding how nurses perceive collegial social support as an important motivational and stimulating factor [40]. Similarly, a quantitative study from Malaysia demonstrates that teamwork and colleagues’ harmony play an important role in nurses’ sense of belongingness in the work culture [41]. Meaningfulness in the workplace is achieved by the joy, togetherness, and security in the working team among nurses in Sweden [16]. Social support from colleagues through solidarity represents a critical job resource needed for reducing job stress and increasing job motivation, performance, and satisfaction among nurses [42, 43]. A better working climate is also associated with significant positive effects on professional commitment and job satisfaction [44]. Therefore, employers and managers should work toward maintaining and promoting an open, sharing, and cooperative work environment in which teamwork, sharing, and mutual support are encouraged.

The result of our study showed that nurses’ barriers to work-related health were a lack of insufficient support from managers and senior-level staff. Nurses experienced that the support was insufficient for fulfilling administrative responsibilities, such as scheduling staff or changing shifts. They also experienced that they were not acknowledged for their work. They were incorrectly evaluated in terms of job performance and promotion reviews. Baral and Subedi [45] presented similar findings, also in Nepal, that nurses’ experience of stress was caused by unfair judgment and lack of good interpersonal relationships with managers. A study from South Korea demonstrates that nurses who have greater managerial support and leadership skills are more likely to feel that their work is meaningful [46]. A qualitative study from Bangladesh also confirmed the adverse impact of a lack of support from nursing supervisors [47]. Job dissatisfaction and burnout among nurses have been related to a lack of good leadership, and it has been argued that adequate skills development for leaders is required to help them develop their emotional leadership skills [47, 48]. Supervisory support and transformational leadership, as well as fair and authentic management, are key job resources for nursing staff, whereas the lack of formal rewards and overload work are key job demands [3]. As experienced by the nurses, sufficient support and fair work evaluations constitute important workplace resources and provide a key motivational factor for mitigating work-related stress. Fair and authentic management, transformational leadership, rewards, and support from the manager are key resources for nursing staff [3].

We found also that most nurses’ barrierstotheir work-related health werestressful and hectic jobs. Nurses experienced that there were staff shortages, severely disproportionate patient-to-nurse ratios, and a lack of basic equipment to protect themselves and for patient care. Fear of contamination, communicable diseases, physical pain symptoms, and lack of recovery were evident as a result of a difficult work environment. Baral and Subedi [45], in recent research in Nepali hospitals, identified that a lack of logistics, high workloads, insufficient staff, and challenging health conditions affected nurses’ work-related health negatively. Recent analytical findings from a quantitative study, also in the context of Nepal, demonstrated a correlation between moderate levels of burnout and stress and time pressure, administrative responsibilities, and dealing with patients’ relatives [49]. Lack of organizational support and heavy workloads among nurses were also found to be evident in the qualitative study in Bangladesh [47]. Managing hectic and stressful work can be challenging for nurses, as they are involved in a profession that requires varying shift schedules. A narrative review by Sun et al. showed that nurses who perform shift work are affected by chronic stress, poor recovery, sleep difficulties, and burnout. The review suggested an increased need for sleep and recovery awareness as well as implementing policies that are supportive for better recovery [50]. Therefore, improving working conditions and the work environment as a whole is crucial for health promotive actions intended to ensure and maintain the good health of nursing personnel as well as to facilitate and sustain high-quality patient care [51]. Early prevention and betterment of the working environment and better recovery strategies in low-income settings such as Nepal should be given a higher priority.

In our study, the nurses also noted some work benefits that facilitated their health, especially medical insurance, and free regular medical checkups. Skills opportunities, such as educational and training courses, were also perceived as benefiting their health, particularly when such training concerning workplace safety practices, for example. Despite this, one recent study revealed that such training and education were unavailable to 74% of the examined nurses [7]. In addition, the nurses had their own coping strategies, such as visiting family and friends, listening to music, meditating, and taking yoga classes, and they employed these to minimize their stress [45]. In summary, job benefits, coping strategies, skills development, education, and training all mitigated the otherwise adverse impacts of workplace stressors, such as high workloads, ultimately reducing stress and enhancing job performance and satisfaction. Supporting the evidence from the job demands–resource model, job resources enhance employees’ job performance, satisfaction, and motivation despite their high work pressures and workloads [21, 22]. We, therefore, recommend that organizations focus on strengthening work resources that nurses find to be facilitating work-related health and that improve their working environment. Investment through such increased resources can be an effective way to improve nurses’ health, well-being, job satisfaction, and work engagement.

### Limitations

We used purposive sampling to reach nurses working in five of the largest hospitals in the Kathmandu Valley. This technique excluded small, private hospitals in addition to several large hospitals outside the valley. Therefore, our results are limited in terms of both their scope and potential for generalizability. Another limitation is that we could not validate our results with the participants, which would have increased the trustworthiness of the findings. Validation of results from the participants in a qualitative study is important and we did attempt to reach the participants via email,to obtain feedback onour results. However, we received only a low number of replies, and in-person meetings were not possible due to the ongoing COVID-19 pandemic.

## Conclusion

Nurses working in hospitals in the Kathmandu Valley, Nepal, experience high workloads and severe stressare barriers totheir workplace health. Therefore requirespersistent, regular, and sufficient attention and investment through measures supportive of their work environment and to sustain their health. Managers and employers should work to improve the physical work environment and ensure the safety of nurses by eliminating physical health hazards and providing the necessary equipment and supplies. It is also important to strengthen nurses’ facilitators for work through regular education classes and skills development opportunities. Managers should also strive to reduce nurse-patient ratios, be more available and approachable to nurses, and be more supportive oftheir everyday work. Moreover, it is vital to identify strategies that will effectively minimize work overload and stress. Common social activities, greater work appreciation, and rewards may help strengthen nurses’ sense of affinity for and meaningfulness in the workplace. Organizational and collegial support is also essential for maintaining and sustaining the health of nurses globally.

## Data Availability

The datasets used and/or analysed during the current study are available from the corresponding author on reasonable request.
